# Evolutionary game analysis of environmental pollution control under the government regulation

**DOI:** 10.1038/s41598-021-04458-3

**Published:** 2022-01-10

**Authors:** Kui Zhou, Qi Wang, Junnan Tang

**Affiliations:** 1 School of Public Finance and Taxation, Zhongnan University of Economics and Law, Wuhan, 430070 China; 2 School of Economics, Fudan University, Shanghai, 200082 China; 3 Central China Securities, Shanghai, 200082 China

**Keywords:** Applied mathematics, Software, Statistics, Environmental sciences, Environmental social sciences, Mathematics and computing

## Abstract

This paper studied a tripartite evolutionary game of stakeholders in environmental pollution control. Most previous studies on this issue are limited to a focus on system dynamics with two-party game problems and lack a spatial analysis of strategy evolution. The parameters adopted are too few, and the influencing factors considered are too simple. The purpose of the paper is to introduce more parameters to study, which will have an important impact on the strategy choices of participants and the evolution path of the strategy over time. We construct a tripartite evolutionary game model of sewage enterprises, governments and the public. We establish a payment matrix and replicator equations as our method, and we also implement parameter simulations in MATLAB. In summary, we found that the reward and punishment mechanism plays an important role in environmental pollution control. Specifically: intensifying rewards and penalties will help encourage sewage enterprises to meet the discharge standard and the public to participate in pollution control action. However, increased rewards will reduce government's willingness to adopt incentive strategies; Government's reward for public's participation in the action must be greater than the increased cost of participation; Reducing the cost of sewage enterprise can also encourage them to implement standard emissions. The research presented in this paper further improves standard emissions and designs reasonable reward and punishment mechanism.

## Introduction

In recent years, with the development of the world's economy, global climate disasters have occurred frequently. The recent floods in Germany and Henan Province of China are all manifestations of climate disasters. Climate disasters are closely related to environmental pollution which poses a threat to us. To control environmental pollution, we need to give full play to the enthusiasm of the government, the media, the public and other stakeholders. In China, due to fiscal decentralization, many polluting enterprises are large local taxpayers. Some governments even act as the "umbrella" for polluting enterprises. The existence of these complex stakeholders makes it difficult to control environmental pollution. Some scholars studied the stakeholders in pollution control. Environmental regulation of the Chinese government has a positive effect on reducing air pollution ^1^. Urban residents, people with high education, high income and social status are more likely to participate in environmental governance^2^. The environmental governance of chemical industrial parks requires multiparty participation^3^. The advantages of dealing with information asymmetry is the logical point of public participation in environmental governance^4^. At present, there are many studies on environmental pollution. However, most of the research focus on the impact and control of environmental pollution^5–9^, while there are relatively few studies on the treatment of environmental pollution from the perspective of stakeholders by using game theory.Evolutionary games are an application of game theory in the field of biological evolution and are now widely used in economics, management and many other aspects. For example, solving the collective-risk social dilemma^10^; optimal institutional incentives^11^; prosocial punishment^12^; incentives for cooperative governance of risky commons^13^; and so on.Smith was the first to study evolutionary games^14^. Later, some scholars extended the model^15,16^to simulate the cooperative evolution of biological populations. Evolutionary game theory holds that the game subjects have bounded rationality, it is difficult to choose the optimal strategy of a single game, and through continuous trial and error and imitation to achieve evolutionary stability^17^. This theory was developed to analyse game players with limited rationality and dynamic games^18–20^. Replicator dynamics identify how pure strategies evolve over time^21^. The most likely outcome of the evolutionary game is determined by the completeness of information obtained by participants and expectations of other participants’ strategies^22,23^.

In the application of game theory to environmental governance, the main research focuses on fully rational subjects. More developed countries can encourage underdeveloped countries to become good "environmental citizens" through direct environmental assistance^24^. The punishment imposed on enterprises in a famous air pollution game model are considered^25,26^. The transnational pollution game shows the Prisoner's dilemma between developing countries^27,28^. Some research has mainly focused on two-party evolutionary games^29–31^. Cooperation between regions leads to increased emission reduction, thereby reducing pollution stocks ^32,33^. When all countries are short-sighted, their benefits are smaller than when all countries are far sighted^34,35^. Countries may respond by increasing emissions, resulting in an increase in pollution stocks^36,37^. A market-oriented regulatory framework is better than inflexible orders and controls^38,39^. There has been a lack of rigorous mathematical demonstration in existing work ^40,41^. Some studies show deficiencies in the impact analysis of parameter changes^42,43^.The greater the incentive of the central government is, the greater the probability of enterprises and local governments choosing environmentally friendly strategies^44,45^, but dynamic analysis is lacking. Only with the help of the superior government can the public play a supervisory role^46,47^. Increasing incentives is conducive to improving the probability of enterprises choosing green innovation^48,49^. If incentives and fines are increased, contractors tend to implement green construction, and the probability of government active supervision is inversely proportional to subsidies and directly proportional to punishment^50,51^. With the increase in rewards and punishments, the time required to achieve stability is becoming increasingly shorter^52,53^.

The purpose of this paper is to design a more practical model to identify the factors that affect the standard emissions of sewage enterprises, the conditions under which reward and publishment mechanism can work, and strategy changes over time and to provide a reference for the reasonable design of standard emission reward and punishment systems. This paper makes the following contributions. (1) We establish replicator equations and draw a diagram of the corresponding strategy to prove the economic rationality of game participants. According to these three-dimensional geometric figures, we use the method of calculus to deduce the volume formula for the government, sewage enterprises and the public to choose corresponding strategy. (2) We use a spatial three-dimensional diagram to show the impact of the change of parameters on the strategy. Using the strategy formula and the method of calculus, we derive the parameters of the formula, and discuss the symbols of these results. Through such rigorous calculus method, it is proved that the conclusion of this paper is more reasonable and reliable. (3) We introduce more parameters, which is more in line with real environments. Many of these parameters are unique to this article, such as Punishment imposed by the superior government for loose supervision, economic compensation for the public's damage. In the part of parameter simulation analysis, the simulation results are also highly consistent with previous mathematical analysis, which proves the rationality and reliability of this conclusion again. (4) We strive to make the results of this paper conform to common principles of economics and prove the rationality of our conclusions through rigorous mathematics. In terms of our method, we establish a tripartite evolutionary game model, a payment matrix of participants and replicator equations, and then we conduct a parameter simulation in MATLAB. How does the reward and punishment mechanism affect the standard emissions of sewage enterprises? What conditions are required for the reward and punishment mechanism to work? Are these conditions applicable to local governments? We will explore these problems in the following section.

## Model assumptions

### Hypothesis and parameter setting

This paper establishes a tripartite evolutionary game model of sewage enterprises, the government and the public. To analyse the existence of the equilibrium point of the evolutionary game and the relationship between various factors, this paper makes the following assumptions on the game:

#### Hypothesis 1

The three players of the game are sewage enterprises, the government and the public. The three parties are bounded rational, so their strategies gradually reach an evolutionarily stable state over time

#### Hypothesis 2

Since sewage enterprises may need to increase the input of technology and personnel to achieve the standard discharge of pollutants, these additional inputs will increase the cost. Due to the pursuit of profit maximization, the input of technology and personnel is short. Therefore, it is necessary for the government to supervise sewage enterprises. The game strategy set of sewage enterprises is $$\updelta = \left( {\updelta _{1} ,\updelta _{2} } \right) = ({\text{Standard emission}},{\text{Excessive emission}})$$, and the sewage enterprise chooses standard emissions with a probability of $$x$$ and excessive emissions with a probability of $${ }1 - x$$. When a pollutant discharging enterprise exceeds the standard, it will be fined $$ F_{e}$$ if it is reported by the public. When the sewage enterprise exceeds the standard, the public can choose to participate in supervision or not. Therefore, the strategic space of the public is as follows:$$ \uplambda = \left( {\uplambda _{1} ,\uplambda _{2} } \right) = ({\text{Participate in supervision}},{\text{Don}}^{^{\prime}} {\text{t}} {\text{participate in}} {\text{supervision}})$$. The probability of the public's election of $$\uplambda _{1}$$ is $$y$$, and the probability of the selection of $$\uplambda _{2}$$ is $$1 - y $$.

Sewage enterprises can bring economic benefits to local governments. On the one hand, due to the existence of China's fiscal decentralization system, sewage enterprises are often large local taxpayers, which can bring considerable financial revenue to local governments and enhance their financial capacity. On the other hand, due to the existence of a central environmental protection supervision system, local governments also have the responsibility to protect the environment. Local governments are facing the dual challenges of economic development and environmental protection. Therefore, local governments may strictly supervise sewage enterprises, but they may also unilaterally pursue GDP and choose loose supervision of polluting enterprises. Therefore, strategies of local governments can be expressed as: ${\upsigma } = \left( {{\upsigma }_{1} ,{\upsigma }_{2} } \right) = \left( {{\text{Strict supervision}},{\text{Loose supervision}}} \right)$. The probability of the government's choice of ${\upsigma }_{1}$ is $z$, and the probability of the choice of ${\upsigma }_{2}$ is $1 - z$.

#### Hypothesis 3

The sales revenue of sewage enterprises is $${\uppi }_{{\text{e}}}$$. In this paper, it is assumed that the sales revenue brought by standard emissions and excessive emissions of sewage enterprises is the same. In a completely competitive market, the assumption that the sales revenue of sewage enterprises is the same is reasonable. The difference between standard emissions and excessive emissions lie in the cost. Due to the need to increase relevant equipment and personnel for standard emissions, the cost of standard emissions are relatively high, while the cost of excessive emissions is relatively low. Therefore, it is assumed that the cost of standard emissions is $$ C_{e1}$$, and the cost of excessive emissions is $$ C_{e2} { }$$,$${ }C_{e1} > C_{e2} { }$$. When a pollutant discharging enterprise exceeds the standard, it will be fined $$ F_{e} { }$$ if it is reported by the public.

#### Hypothesis 4

The strategy of the public is to choose to participate in supervision and not participate in supervision. Suppose that the government's reward to the public involved in supervision is $$ R_{P}$$. It is assumed that only when the government chooses to strictly supervise sewage enterprises will the public be rewarded for participating in supervision; otherwise, when the government chooses loose supervision, no reward will be given. When the public participates in supervision, they will pay a certain supervision cost, assuming that the cost is $${ }C_{P}$$. The damage caused to the public by excessive pollutant discharge by enterprises is $${ }H_{P}$$. The economic compensation obtained from the sewage enterprise is $$ S_{P}$$.

#### Hypothesis 5

The economic benefit brought by the development of sewage enterprises to local governments is $$ \pi_{g}$$, and the government's strategy is to conduct strict and loose supervision of sewage enterprises. When the governments choose strict supervision, if the pollutant discharge enterprise exceeds the standard, it will be fined with an amount of $$ F_{e}$$; when local governments choose strict supervision, they will reward the public $$ R_{P} { }$$ participating in the supervision. When the government chooses loose supervision, it will not reward and punish sewage enterprises and the public. Suppose the cost of strict regulation is $$C_{g1}$$.When the government chooses strict supervision, it will strictly restrict the environmental violations of enterprises, which may hinder the development of local economies. This part of the potential losses will be recorded as $$ L$$. If the local government chooses loose regulation, its cost is assumed to be $$ C_{g2}$$. As we know,$${ }C_{g1} > C_{g2}$$.When the loose supervision of government departments leads to the excessive discharge of sewage enterprise, they will be punished by the superior government, assuming that the amount of punishment is $$ F_{g}$$, Table 1 lists relevant parameters of the tripartite evolutionary game.

**Table 1:** Relevant parameters of the evolutionary game model.

Parameter	Explanation of parameter's meaning
${\uppi }_{{\text{e}}}$	Sales revenue of sewage enterprise
$C_{e1}$	Cost of standard emission of sewage enterprises
$C_{e2}$	Cost of excessive emission of sewage enterprises
$$ F_{e}$$	Fines imposed by the government for excessive emission
$$ R_{P}$$	Reward for the public participating in the supervision
$C_{P}$	Supervision cost paid by the public when participating in supervision
$H_{P}$	Damage to the masses caused by excessive pollutant discharge
$$ S_{P}$$	Economic compensation for the public's damage
$\pi_{g}$	Benefits brought by the development of sewage enterprises to government
$C_{g1}$	The cost of strict government regulation
$C_{g2}$	The cost of loose government regulation
$L$	Potential economic losses caused by strict government supervision
$$ F_{g}$$	Punishment imposed by the superior government for loose supervision

### Establishment of the model

This paper establishes a tripartite evolutionary game model of sewage enterprises, governments and the public. By analysing the strategies of all parties involved in the game, the following game payment matrix is established, as shown in Table 2.

**Table 2:** Tripartite evolutionary game payment matrix of the model.

Public	Local government	
	Strict supervision $z$	Loose supervision $1 - z$
Sewage enterprises		
Standard emission $x$		
Participate in supervision $y$	$\uppi _{e} - C_{e1}$	$\uppi _{e} - C_{e1}$
	$$ R_{P} - C_{P}$$	$$- C_{P}$$
	$\pi_{g} - L - C_{g1} - R_{P}$	$\pi_{g} - C_{g2}$
Don't participate in supervision $1 - y$	$\uppi _{e} - C_{e1}$	$\uppi _{e} - C_{e1}$
	0	0
	$\pi_{g} - L - C_{g1}$	$\pi_{g} - C_{g2}$
Excessive emission 1-$x$		
Participate in Supervision $y$	$\uppi _{e} - C_{e2} - F_{e} - S_{P}$	$\uppi _{e} - C_{e2} - F_{e} - S_{P}$
	$$ R_{P} - C_{P} + S_{P} - H_{P}$$	$$- C_{P} + S_{P} - H_{P}$$
	$\pi_{g} - L - C_{g1} - R_{P} + F_{e}$	$\pi_{g} - C_{g2} - F_{g} + F_{e}$
Don't participate in supervision $1 - y$	$\uppi _{e} - C_{e2} - F_{e} - S_{P}$	$\uppi _{e} - C_{e2}$
	$$ S_{P} - H_{P}$$	$$- H_{P}$$
	$\pi_{g} - L - C_{g1} + F_{e}$	$\pi_{g} - C_{g2}$

## Model analysis

### Evolutionary equilibrium strategy analysis of standard emissions of sewage enterprises

It is assumed that the expected income of standard emissions is $$ E_{11}$$,, the expected income of excessive emissions is $$ E_{12}$$, and the total average expected income is $$E_{1}$$.

1 $$ \left\{ \begin{gathered} E_{11} = yz\left( { \pi_{e} - C_{e1} } \right) + y\left( {1 - z} \right)\left( { \pi_{e} - C_{e1} } \right) + \left( {1 - y} \right)z\left( { \pi_{e} - C_{e1} } \right) \hfill \\ \quad + \left( {1 - y} \right)\left( {1 - z} \right)\left( { \pi_{e} - C_{e1} } \right) = \pi_{e} - C_{e1} \hfill \\ E_{12} = yz\left( { \pi_{e} - C_{e2} - F_{e} - S_{P} } \right) + y\left( {1 - z} \right)\left( {\pi_{e} - C_{e2} - F_{e} - S_{P} } \right) \hfill \\ \quad + \left( {1 - y} \right)z\left( { \pi_{e} - C_{e2} - F_{e} - S_{P} } \right) + \left( {1 - y} \right)\left( {1 - z} \right)\left( {\pi_{e} - C_{e2} } \right) \hfill \\  \quad= \pi_{e} - C_{e2} - \left( { F_{e} + S_{P} } \right)\left[ {y + \left( {1 - y} \right)z} \right] \hfill \\ E_{1} = x E_{11} + \left( {1 - x} \right) E_{12} \hfill \\ \end{gathered} \right. $$

The replicator dynamic equation of sewage enterprises is as follows:

2 $$ F\left( x \right) = \frac{dx}{{dt}} = x\left( {E_{11} - E_{1} } \right) = x\left( {1 - x} \right)\left[ {C_{e2} - C_{e1} + y\left( {1 - y} \right)z\left( { F_{e} + S_{P} } \right)} \right] $$

The first derivative of $F\left( x \right)$ with respect to $x$ is as follows:

3 $$ \frac{{\partial (F\left( {x)} \right)}}{\partial x} = \left( {2x - 1} \right)\left[ {C_{e1} - C_{e2} - y\left( {1 - y} \right)z\left( { F_{e} + S_{P} } \right)} \right] $$

4 $$ {\text{Let }}H\left( z \right) = C_{e1} - C_{e2} - y\left( {1 - y} \right)z\left( { F_{e} + S_{P} } \right) $$

**Figure 1:**
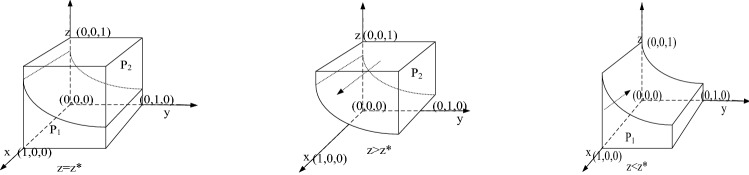
Strategic diagram of sewage enterprises.

According to the stability principle of the differential equation, the probability of standard emissions by sewage enterprise $$x$$ needs to meet the following condition to reach steady state: $$F\left( x \right) = 0,\frac{{\partial (F\left( {x)} \right)}}{\partial x} < 0$$. Because $$\frac{{\partial \left( {H\left( z \right)} \right)}}{\partial \left( z \right)} = - y\left( {1 - y} \right)\left( { F_{e} + S_{P} } \right)$$ and coefficient $$ F_{e} + S_{P} > 0$$ such that $$\frac{{\partial \left( {H\left( z \right)} \right)}}{\partial \left( z \right)} < 0$$ and $$H\left( z \right)$$ is a subtraction function of $$z$$. When $$z = \frac{{C_{e1} - C_{e2} }}{{y\left( {1 - y} \right)\left( { F_{e} + S_{P} } \right)}} = z^{*}$$, $$H\left( z \right) = 0$$,and $$F\left( x \right) \equiv 0$$, then all $$x$$ values will make the sewage enterprise be in an evolutionarily stable state. When $$z < z^{*}$$, $$H\left( z \right) > 0$$, $$x = 0$$ is the evolutionary game stable strategy of sewage enterprises. In contrast, when $$ z > z^{*}$$, $$H\left( z \right) < 0$$, and $$x = 1$$ is the evolutionary game stable strategy of sewage enterprises; that is, when the probability of strict supervision by the government is high, sewage enterprises tend to meet the discharge standard. The diagram of the evolutionary game of pollutant discharge is shown in Fig. 1.

Figure 1 shows that the evolutionary game stable probability of sewage enterprises choosing standard emissions is the volume shown in part *P*_*2*_, and we use $V_{{P_{2} }}$ to express it. This indicates that it was the evolutionary game stable strategy of sewage enterprises at that time. In other words, the selection of standard emissions are the evolutionary strategy of sewage enterprises. The volume of the probability of standard emission is $V_{{P_{2} }}$, which can be calculated as follows:

$$ V_{{P_{1} }} = \mathop \smallint \limits_{0}^{1} \mathop \smallint \limits_{0}^{1} \frac{{C_{e1} - C_{e2} }}{{y\left( {1 - y} \right)\left( { F_{e} + S_{P} } \right)}}dydx = { }\frac{{C_{e1} - C_{e2} }}{{\left( { F_{e} + S_{P} } \right) }},\;\;V_{{P_{2} }} = 1 - V_{{P_{1} }} $$

*Inference 1*: The probability of standard emissions has a negative relationship with the cost of standard emissions $$C_{e1}$$; that is, the higher the cost of standard emissions is, the more unfavourable it is for the sewage enterprise to meet the emission standard. The probability of standard emissions has a positive relationship with economic compensation for the public's damage $$ S_{P} { }$$ and fines imposed by the government for excessive emissions $$ S_{P}$$.

We will show it: according to the probability formula of standard emission of pollutant discharge enterprise $$V_{{P_{2} }}$$, we can get the first derivative of $$ V_{{P_{2} }}$$ with respect to the cost of standard emission $$ C_{e1}$$, economic compensation for the public's damage $$S_{P}$$, penalty for excessive emissions $$ F_{e}$$:

$$ \partial V_{{P_{2} }} /\partial C_{e1} < 0,{ }\partial V_{{P_{2} }} /\partial S_{P} > 0{ },{ }\partial V_{{P_{2} }} /\partial F_{e} > 0 $$

Therefore, the probability of standard emissions has a negative relationship with the cost of standard emissions $$ C_{e1}$$; that is, the higher the cost of standard emissions is, the more unfavourable it is for sewage enterprises to meet the standard emissions. The probability of standard emissions has a positive relationship with economic compensation for public damage $$ S_{P} { }$$ and government punishment $$ F_{e}$$ for sewage enterprises that fail to meet discharge standards. The reduction and increase in these parameters will improve the probability of standard emission. The government should strengthen policy stimulation to increase punishment for excessive emissions, improve the income of sewage enterprises, and reduce the cost of standard emissions to improve the enthusiasm of sewage enterprises to implement standard emissions.

Inference 2: The probability of sewage enterprises reaching the standard $$x$$ is negatively correlated with the probability of strict government supervision $$ z$$. When $$z < z^{*}$$, $$H\left( z \right) > 0$$, $$x = 0$$ is the evolutionarily stable strategy of sewage enterprises. In contrast, when $$z > z^{*}$$, $$H\left( z \right) < 0$$, and $$x = 1$$ is the evolutionarily stable strategy of sewage enterprises, which shows that the greater the punishment of the government on sewage enterprises, the greater the probability of sewage enterprises choosing to meet the discharge standard. This shows that the government needs to strengthen restrictive policies and promote sewage enterprises to meet discharge standards.

### Analysis on evolutionary equilibrium strategy of the public's supervisory participation

Assuming that the expected return of public participation in the supervision of sewage enterprises is $E_{21}$, the expected return of no participation in supervision is $E_{22}$, and the total average expected return is $E_{2}$ .

5 $$ \left\{ \begin{array}{*{20}c} E_{21} = xz\left( { R_{P} - C_{P} } \right) + x\left( {1 - z} \right)\left( { - C_{P} } \right) + \left( {1 - x} \right)z\left( { R_{P} - C_{P} + S_{P} - H_{P} } \right)  \hfill \\ \qquad +\left( {1 - x} \right)\left( {1 - z} \right)\left( { - C_{P} + S_{P} - H_{P} } \right) = zR_{P} - C_{P} + \left( {1 - x} \right)\left( { S_{P} - H_{P} } \right) \hfill \\ E_{22} = \left( {1 - x} \right)z\left( { S_{P} - H_{P} } \right) + \left( {1 - x} \right)\left( {1 - z} \right)\left( { - H_{P} } \right) = \left( {1 - x} \right)\left( {zS_{P} - H_{P} } \right) \hfill \\ E_{2} = yE_{21} + \left( {1 - y} \right)E_{22} \hfill \\ \end{array} \right. $$

The replicator dynamic equation of public participation in the supervision of sewage enterprises is as follows:

6 $$ \begin{aligned} F\left( y \right) &=  \frac{dy}{{dt}} = y\left( {E_{21} - E_{2} } \right) = y\left( {1 - y} \right)\left( { E_{21} - E_{22} } \right) \\ & = y\left( {y - 1} \right)\left[ {C_{P} - zR_{P} - \left( {1 - x} \right)\left( {1 - z} \right)S_{P} } \right] \\ \end{aligned} $$

The first derivative of the replicator dynamic equation of public participation in supervision is:

7 $$ \frac{{\partial \left( {F\left( y \right)} \right)}}{\partial y} = \left( {2y - 1} \right)\left[ {C_{P} - zR_{P} - \left( {1 - x} \right)\left( {1 - z} \right)S_{P} } \right] $$

8 $$ {\text{Let}}\;G\left( x \right) = C_{P} - zR_{P} - \left( {1 - x} \right)\left( {1 - z} \right)S_{P} $$

**Figure 2:**
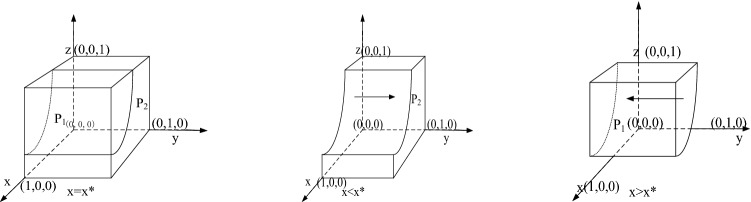
Strategy diagram of the public.

According to the stability principle of the differential equation, the probability of public participation in supervision needs to meet the following conditions:$F\left( y \right) = 0$,${ }\frac{{\partial \left( {F\left( y \right)} \right)}}{\partial y} < 0$. Because $\frac{{{ }\partial \left( {G\left( x \right)} \right)}}{{\partial \left( {\text{x}} \right)}} = \left( {1 - z} \right)S_{P}$ and the economic compensation given to the public is positive, $G\left( x \right)$ is an increasing function of *x*. When $x = \frac{{S_{P} - C_{P} + z\left( {R_{P} - S_{P} } \right)}}{{\left( {1 - z} \right)S_{P} }} = x^{*}$, all ${ }x$ values will make the public in an evolutionarily stable state. When $x < x^{*}$, $G\left( x \right) < 0$, and $y = 1$ is the evolutionary game stable strategy of the public. In contrast, when ${ } x > x^{*}$, $G\left( x \right) > 0$,${ }y = 0$ is the evolutionarily stable strategy of the public; that is, when the probability of sewage enterprises choosing standard emissions was low, the public tended to participate in the supervision of sewage enterprises. The evolutionary game diagram of the public is shown in Fig. 2.

Figure 2 shows that the evolutionary probability of public participation in the supervision of sewage enterprises can be expressed as the volume of part $P_{2}$. We use $V_{{P_{2} }} { }$ to express it. When $x < x^{*}$, $y = 1$ is the stable state of the public. In other words, participation in the supervision of sewage enterprises is the evolutionarily stable strategy of the public. Through the calculation, we can get $V_{{P_{2} }}$:

$$ V_{{P_{2} }} = \mathop \smallint \limits_{0}^{1} \mathop \smallint \limits_{0}^{1} \frac{{S_{P} - C_{P} + z\left( {R_{P} - S_{P} } \right)}}{{\left( {1 - z} \right)S_{P} }}dzdy = \frac{{S_{P} - C_{P} + R_{P} }}{{S_{P} }},{ }V_{{P_{1} }} = 1 - V_{{P_{2} }} $$

*Inference 3*: The probability of public participation in the supervision of sewage enterprise $y$ is negatively correlated with the cost of participation in supervision ${ }C_{P}$ , but it is positively correlated with the government's reward for public participation in supervision $R_{P}$.

According to the probability formula of the probability of public participation in the supervision of sewage enterprise $V_{{P_{2} }}$, the first-order derivation of the formula on the cost of public participation in supervision $C_{P}$ and government reward for public participation in supervision $R_{P}$ can be obtained:

$$\partial V_{{P_{2} }} /\partial C_{p} < 0 ,{ }\partial V_{{P_{2} }} /\partial R_{P} > 0{ }$$,

Therefore, an increase in the government's reward for participating in the supervision of sewage enterprises $R_{P}$ will improve the probability of public participation in the supervision of sewage enterprises. An increase in the cost of public participation in supervision $C_{P}$ will reduce the probability of public participation in the supervision of sewage enterprises.

*Inference 4*: The probability of public participation in the supervision of sewage enterprise $$y$$ is negatively correlated with the probability of sewage enterprise selection of standard emissions. When $$x < x^{*} { }$$ and $$ G\left( x \right) < 0$$, $$y = 1 $$ is the public's evolutionarily stable strategy. In contrast, when $$x > x^{*}$$, $$G\left( x \right) > 0 $$, $${ }y = 0$$ is the public’s evolutionarily stable strategy. $$\frac{{\partial \left( {G\left( {x,z} \right)} \right)}}{\partial \left( x \right)} > 0$$ indicates that the smaller the probability of sewage enterprises choosing standard emissions are, the greater the probability of the public participating in the supervision of sewage enterprises. Therefore, the inaction of sewage enterprises force the public to participate in supervision.

### Analysis of the evolutionary equilibrium strategy of government

Assuming that the expected return of the government's strict supervision of sewage enterprises is $E_{31}$, the expected return of loose supervision is $E_{32}$, and the total average expected return is $E_{3}$.

9 $$ \left\{ \begin{array}{*{20}c} E_{31} = xy\left( { \pi_{g} - L - C_{g1} - R_{P} } \right) + x\left( {1 - y} \right)( \pi_{g} - L - C_{g1} )  \hfill \\ \qquad +\left( {1 - x} \right)y\left( { \pi_{g} - L - C_{g1} - R_{P} + F_{e} } \right) + \left( {1 - x} \right)\left( {1 - y} \right)\left( { \pi_{g} - L - C_{g1} + F_{e} } \right) \hfill \\ \qquad = \pi_{g} - L - C_{g1} + \left( {1 - x} \right) F_{e} - yR_{P} \hfill \\ E_{32} = xy\left( { \pi_{g} - C_{g2} } \right) + x\left( {1 - y} \right)\left( { \pi_{g} - C_{g2} } \right) + \left( {1 - x} \right)y\left( { \pi_{g} - C_{g2} - F_{g} + F_{e} } \right)  \hfill \\ \qquad +\left( {1 - x} \right)\left( {1 - y} \right)\left( { \pi_{g} - C_{g2} } \right) = \pi_{g} - C_{g2} + \left( {1 - x} \right)y\left( { F_{e} - F_{g} } \right) \hfill \\ E_{3} = zE_{31} + \left( {1 - z} \right)E_{32} \hfill \\ \end{array} \right. $$

Government's replicator dynamic equation is:

10 $$ \begin{aligned} F\left( z \right) &=  \frac{dz}{{dt}} = z\left( {E_{31} - E_{3} } \right) = z\left( {1 - z} \right)\left( {E_{31} - E_{32} } \right) \\ & = z\left( {z - 1} \right)\left[ {L + C_{g1} - C_{g2} - \left( {1 - x} \right) F_{e} + yR_{P} + \left( {1 - x} \right)y\left( { F_{e} - F_{g} } \right)} \right] \\ \end{aligned} $$

The first derivative of $F\left( z \right)$ with respect to $z$ is:

11 $$ \frac{{\partial \left( {F\left( z \right)} \right)}}{\partial z} = \left( {2z - 1} \right)\left[ {L + C_{g1} - C_{g2} - \left( {1 - x} \right) F_{e} + yR_{P} + \left( {1 - x} \right)y\left( {F_{e} - F_{g} } \right)} \right] $$

12 $$ {\text{Let }}J\left( y \right) = \left[ { L + C_{g1} - C_{g2} - \left( {1 - x} \right) F_{e} + yR_{P} + \left( {1 - x} \right)y\left( {F_{e} - F_{g} } \right)} \right] $$

**Figure 3:**
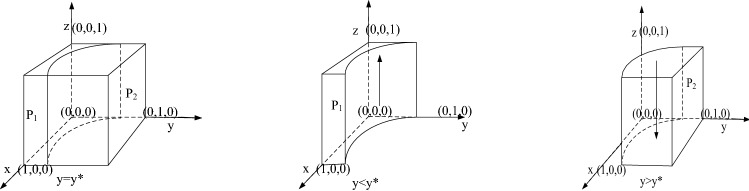
Diagram of the government's strategy.

According to the stability principle of the differential equation, the probability of the government's regulation reaching steady state needs to meet the following conditions:$$ F\left( z \right) = 0$$,$${ }\frac{{\partial \left( {F\left( z \right)} \right)}}{\partial z} < 0$$. Because $${ }\frac{{\partial \left( {J\left( y \right)} \right)}}{\partial y} > 0$$,so $$ J\left( y \right)$$ is an increasing function of $$ y$$. Therefore, when $$y = \frac{{\left( {1 - x} \right) F_{e} + C_{g2} - C_{g1} - L}}{{R_{P} + \left( {1 - {\text{x}}} \right)\left( {F_{e} - F_{g} } \right)}} = y^{*}$$, $$J\left( y \right) = 0{ }$$, and $$F\left( z \right) \equiv 0$$, all $$z$$ values will make the government in an evolutionarily stable state. When $$y < y^{*}$$, $$J\left( y \right) < 0$$, and $$z = 1$$ is the stable state of the government. In contrast, when $$y > y^{*}$$, $$J\left( y \right) > 0$$, $$z = 0$$ is the stable state of the government; that is, when the probability of public participation in the supervision of sewage enterprises was low, the government would tend to implement strict regulatory policy. The diagram of the government's evolutionary game is shown in Fig. 3.

Figure 3 shows that the evolutionarily stable probability of government regulation can be expressed in the volume of part $$P_{1}$$, and we use $${ }V_{{P_{1} }}$$ to express it. When $$y < y^{*}$$, $$z = 1 $$ is the stable state of the government. In other words, strict supervision of sewage enterprises are the evolutionarily stable strategy of the government. The probability of government can be calculated as follows:

$$ V_{{P_{1} }} = \mathop \smallint \limits_{0}^{1} \mathop \smallint \limits_{0}^{1} \frac{{\left( {1 - x} \right) F_{e} + C_{g2} - C_{g1} - L}}{{R_{P} + \left( {1 - {\text{x}}} \right)\left( {F_{e} - F_{g} } \right)}}dxdz $$

*Inference 5*: The probability of government strict supervision of sewage enterprises is positively correlated with fines on sewage enterprises $$ F_{e} { }$$ and penalties imposed by the superior government on the subordinate government $$ F_{g}$$, but it is negatively correlated with the cost of strict supervision $$C_{g1} { }$$ and rewards for public participation in supervision $$R_{P}$$.According to the probability formula of government strict supervision, the first-order derivatives of $$ F_{e}$$,$${ } F_{g}$$, $${ }C_{g1}$$ and $$R_{P}$$ can be obtained:

$$ \partial V_{{P_{1} }} /\partial F_{e} > 0,{ }\partial V_{{P_{1} }} /\partial F_{g} > 0{ },{ }\partial V_{{P_{1} }} /\partial C_{g1} < 0 ,{ }\partial V_{{P_{1} }} /\partial R_{P} < 0 $$

Therefore, the increase in the penalty for sewage enterprises and the penalty for loose supervision of local governments by superior governments will improve the probability of government strict supervision. However, the first derivative of $C_{g1}$ and $R_{P} { }$ is less than 0, indicating that the increase of the cost of strict supervision and reward to the people participating in supervision will reduce government's willingness to implement strict regulatory policy, which shows that with the increase of the cost of government's strict regulatory policy, government's enthusiasm for strict supervision will be reduced.

*Inference 6*: There is a negative correlation between the probability of the government implementing strict regulatory policy $z$ and the probability of the public participating in the supervision of sewage enterprises $y$. When $y < y^{*}$, $J\left( y \right) < 0{ }$, $z = 1$ is the stable state of the government. In contrast, when $y > y^{*}$, $J\left( y \right) > 0$, $z = 0$ is the stable state of the government; that is, when the probability of public participation in the supervision of sewage enterprises is low, the government tends to implement strict supervision policies. $\frac{{\partial \left( {J\left( y \right)} \right)}}{\partial y} > 0$ indicates that the greater the probability of the public's participation in the supervision, the smaller the probability of the government formulating regulatory policy. In contrast, the smaller the probability of public participation in the supervision of sewage enterprises, the greater the probability of the government formulating strict regulatory policies.

### Stable state analysis of the tripartite evolutionary game

Here, we use the dynamic equilibrium of the evolutionary game and Lyapunov's methody^54^ to study the possible equilibrium points of the following three differential equations: (2), (6), and (10).

According to $F\left( x \right) = 0$,${ }F\left( y \right) = 0$, $F\left( z \right) = 0$, the equilibrium point of the tripartite evolutionary game can be obtained as follows:

$$ \left\{ \begin{gathered} F\left( x \right) = \frac{dx}{{dt}} = x\left( {E_{11} - E_{1} } \right) = x\left( {1 - x} \right)\left[ {C_{e2} - C_{e1} + y\left( {1 - y} \right)z\left( { F_{e} + S_{P} } \right)} \right] = 0 \hfill \\ F\left( y \right) = \frac{dy}{{dt}} = y\left( {E_{21} - E_{2} } \right) = y\left( {1 - y} \right)\left( { E_{21} - E_{22} } \right) \hfill \\ \; = y\left( {y - 1} \right)\left[ {C_{P} - zR_{P} - \left( {1 - x} \right)\left( {1 - z} \right)S_{P} } \right] = 0 \hfill \\ F\left( z \right) = \frac{dz}{{dt}} = z\left( {E_{31} - E_{3} } \right) = z\left( {1 - z} \right)\left( {E_{31} - E_{32} } \right) \hfill \\ \; = z\left( {z - 1} \right)\left[ {L + C_{g1} - C_{g2} - \left( {1 - x} \right) F_{e} + yR_{P} + \left( {1 - x} \right)y\left( { F_{e} - F_{g} } \right)} \right] = 0 \hfill \\ \end{gathered} \right. $$

Eight equilibrium points can be obtained by solving the following equations:

$$E_{1} \left( {0,0,0} \right) $$, $$E_{2} \left( {1,0,0} \right) ,E_{3} \left( {0,1,0} \right) ,E_{4} \left( {0,0,1} \right) ,E_{5} \left( {1,1,0} \right) ,E_{6} \left( {1,0,1} \right) ,E_{7} \left( {0,1,1} \right) ,E_{8} \left( {1,1,1} \right)$$.

The Jacobian matrix of the tripartite evolutionary game is:

$$ \begin{aligned} & J = \left[ {\begin{array}{*{20}c} {a_{11} } & {a_{12} } & {a_{13} } \\ {a_{21} } & {a_{22} } & {a_{23} } \\ {a_{31} } & {a_{32} } & {a_{33} } \\ \end{array} } \right] = \left[ {\begin{array}{*{20}c} {\frac{{\partial (F\left( {x)} \right)}}{\partial x}} & {\frac{{\partial (F\left( {x)} \right)}}{\partial y}} & {\frac{{\partial (F\left( {x)} \right)}}{\partial z}} \\ {\frac{{\partial (F\left( {y)} \right)}}{\partial x}} & {\frac{{\partial (F\left( {y)} \right)}}{\partial y}} & {\frac{{\partial (F\left( {y)} \right)}}{\partial z}} \\ {\frac{{\partial (F\left( {z)} \right)}}{\partial x}} & {\frac{{\partial (F\left( {z)} \right)}}{\partial y}} & {\frac{{\partial (F\left( {z)} \right)}}{\partial z}} \\ \end{array} } \right] \\ & = \left[ {\begin{array}{*{20}c} {\left( {2x - 1} \right)\left[ {C_{e2} - C_{e1} - y\left( {1 - y} \right)z\left( {F_{e} + S_{P} } \right)} \right]} & {x\left( {x - 1} \right)\left( {2y - 1} \right)z\left( {F_{e} + S_{P} } \right)} & {xy\left( {x - 1} \right)\left( {y - 1} \right)\left( {F_{e} + S_{P} } \right)} \\ {y\left( {y - 1} \right)\left( {1 - z} \right) S_{P} } & {\left( {2y - 1} \right)\left[ {C_{P} - zR_{P} - \left( {1 - x} \right)\left( {1 - z} \right)S_{P} } \right]} & {y\left( {y - 1} \right)\left[ { - R_{P} + \left( {1 - x} \right) S_{P} } \right]} \\ {z\left( {z - 1} \right)\left[ {F_{e} - y\left( {F_{e} - F_{g} } \right)} \right]} & {z\left( {z - 1} \right)\left[ {R_{P} + \left( {1 - x} \right)\left( {F_{e} - F_{g} } \right)} \right]} & {\left( {2z - 1} \right)\left[ {L + C_{g1} - C_{g2} - \left( {1 - x} \right) F_{e} + yR_{P} + \left( {1 - x} \right)y\left( { F_{e} - F_{g} } \right)} \right]} \\ \end{array} } \right] \\ \end{aligned} $$

The calculated $$E_{1} - E_{8} $$ points are substituted into the above Jacobian matrix to obtain the characteristic matrix corresponding to these points, and the stable state of the evolutionary game needs to meet the condition that eigenvalues of the Jacobian matrix are nonpositive numbers. Taking the equilibrium point $$E_{1} { }$$ as an example, the Jacobian matrix corresponding to this point is:

$$ \left[ {\begin{array}{*{20}c} {C_{e2} - C_{e1} } & 0 & 0 \\ 0 & {S_{p} - C_{p} } & 0 \\ 0 & 0 & {F_{e} + C_{g2} - L - C_{g1} } \\ \end{array} } \right] $$

Three eigenvalues can be obtained from the matrix:

$$ \lambda_{1} = C_{e2} - C_{e1} ,\lambda_{2} = S_{p} - C_{p} ,\lambda_{3} = F_{e} + C_{g2} - L - C_{g1} $$

Because the cost of standard emission is greater than the cost of excessive emission, the symbols of the other two eigenvalues cannot be determined, so it is impossible to determine whether it is a stable point of the tripartite evolutionary game. The eigenvalues corresponding to all eight equilibrium points are shown in Table 3.

**Table 3:** Eigenvalues of the Jacobian matrix corresponding to each equilibrium point.

Equilibrium point	Eigenvalue 1	Eigenvalue 2	Eigenvalue 3
$$E_{1} \left( {0,0,0} \right)$$	$$C_{e2} - C_{e1}$$	$$ S_{P} - C_{p}$$	$$F_{e} + C_{g2} - L - C_{g1}$$
$E_{2} \left( {1,0,0} \right)$	$C_{e2} - C_{e1}$	$zR_{P} - C_{p}$	$C_{g2} - L - C_{g1}$
$E_{3} \left( {0,1,0} \right)$	$C_{e2} - C_{e1}$	$C_{p} - S_{P}$	$C_{g2} - L - C_{g1} - R_{P} + F_{g}$
$E_{4} \left( {0,0,1} \right)$	$C_{e2} - C_{e1}$	$R_{P} - C_{p}$	$C_{g1} + L - C_{g2} - F_{e}$
$E_{5} \left( {1,1,0} \right)$	$C_{e2} - C_{e1}$	$C_{p}$	$C_{g2} - C_{g1} - L - R_{P}$
$E_{6} \left( {1,0,1} \right)$	$C_{e2} - C_{e1}$	$R_{P} - C_{p}$	$C_{g1} + L - C_{g2}$
$E_{7} \left( {0,1,1} \right)$	$C_{e2} - C_{e1}$	$C_{p} - { }R_{P}$	$C_{g1} + L - C_{g2} + R_{P} - F_{g}$
$E_{8} \left( {1,1,1} \right)$	$C_{e2} - C_{e1}$	$C_{p} - { }R_{P}$	$C_{g1} + L - C_{g2} + R_{P}$

After analysing the stable point of the evolutionary game, conditions for the existence of a stable state of are given. The results are shown in Table 4:

**Table 4:** Stability analysis of equilibrium point.

Equilibrium point	Eigenvalue symbol	Stability	Condition
$E_{1} \left( {0,0,0} \right)$	$\left( { - , + , \times } \right)$	Unstable point	\
$E_{2} \left( {1,0,0} \right)$	${ }\left( { - , + , \times } \right)$	Unstable point	\
$E_{3} \left( {0,1,0} \right)$	${ }\left( { - , - , - } \right)$	ESS	①
$E_{4} \left( {0,0,1} \right)$	${ }\left( { - , + , \times } \right)$	Unstable point	\
$E_{5} \left( {1,1,0} \right)$	${ }\left( { - , + , \times } \right)$	Unstable point	\
$E_{6} \left( {1,0,1} \right)$	${ }\left( { - , + , \times } \right)$	Unstable point	\
$E_{7} \left( {0,1,1} \right)$	${ }\left( { - , - , - } \right)$	ESS	②
$E_{8} \left( {1,1,1} \right)$	${ }\left( { - , - , - } \right)$	ESS	③

x indicates uncertain symbol, condition:① $$C_{g2} - L - C_{g1} - R_{P} + F_{g} < 0 $$, ②:$${ }C_{g1} + L - C_{g2} + R_{P} - F_{g} < 0 $$, ③: $$C_{g1} + L - C_{g2} + R_{P} < 0$$

Inference 7: when conditions ①, ②, and ③ are satisfied, there are three equilibrium points $E_{3} \left( {0,1,0} \right){ }$,$E_{7} \left( {0,1,1} \right)$,and $E_{8} \left( {1,1,1} \right)$. This shows that the government's stable strategy is to adopt a loose regulatory policy when the sewage enterprise chooses excessive emissions and the public chooses to participate in the supervision of sewage enterprises.

## Simulation analysis

In this section, the parameters of the model are assigned based on the replicator dynamic equation: (2), (6),and (10).Relevant statistical data from the China Environmental Statistics Yearbook, China Statistical Yearbook and the relevant data of a sewage company in Zhejiang Province are used. On April 8, 2021, a sewage company discharged wastewater from an aluminium product processing points in Pingyang County, Wenzhou City, Zhejiang Province, which was reported to the Pingyang branch of the Wenzhou Ecological Environment Bureau by the public. After sampling, the law enforcement officers of the Bureau found that the content of heavy metals such as copper, chromium and zinc in the wastewater exceeded the national pollutant discharge standard. Pingyang branch awarded the informant 10,000 yuan.Since the values of some variables are interval values, their average value is used for convenience. We use MATLAB for our following analysis:

$$ C_{e1} = 53,{ }C_{e2} = 26,{ } S_{P} = 133,{ }F_{e} = 38,{ }C_{p} = 2.3 $$

$$ { }R_{P} = 4.1,{ }C_{g1} = 37,{ }C_{g2} = 17.4, L = 13.7,{ }F_{g} = 6.1 $$

Assuming that in the initial state, the corresponding probability value selected by the three parties is $$x = 0.5;y = 0.5;z = 0.5 $$, the influence of the change in each parameter on the probability of the strategy selection is analysed.

### Effect of changes in emission costs of sewage enterprise

**Figure 4:**
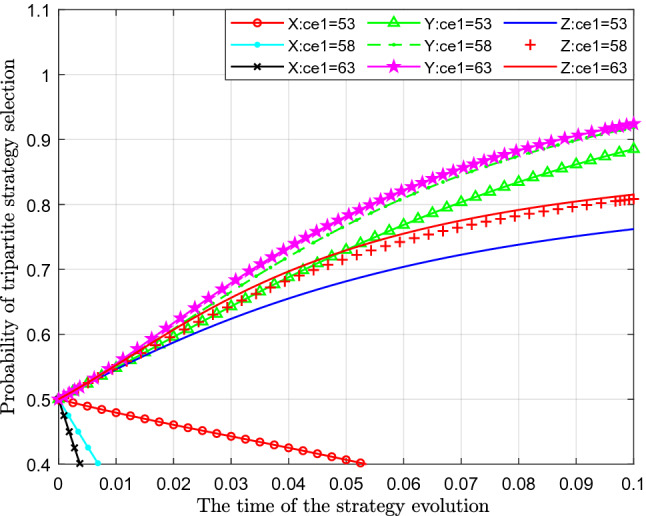
Effect of the change in the standard emission cost of sewage enterprises.

We have studied the impact of the change of emission cost of sewage enterprise on the strategic choice of government, sewage enterprise and the public. Figure 4 analyses the impact of these changes on the strategy. This paper assigns values of 53, 58 and 63 to $C_{e1}$ for analysis. The horizontal axis represents the time of the simulation of the strategy evolution, and the vertical axis represents the probability of selection of the corresponding strategy.

Figure 4 shows the effect of the change in the cost of standard emissions. From the figure, we can see that with the continuous increase of the value of standard emission, the probability of standard emission $$X $$ represented by separate colour curves are decreasing, and its probability is gradually approaching 0.4. At the same time, the time to reach 0.4 becomes increasingly shorter. From the perspective of the probability of public participation in the supervision of sewage enterprises $$Y$$, with the increase in the cost of standard emissions, the probability of public participation in the supervision of sewage enterprises has increased significantly, which can be seen in the figure that the curves move upwards by a large margin. From the perspective of the impact on the probability of government strict supervision $$Z$$, with a continuous increase in the cost of standard emissions, the probability of government strict supervision also increases significantly. This shows that an increase in standard emissions leads to a decrease in the probability of standard emissions, but it increases the probability of strict supervision and public participation in supervision.

### Effect of change of excessive emission cost of sewage enterprise

**Figure 5:**
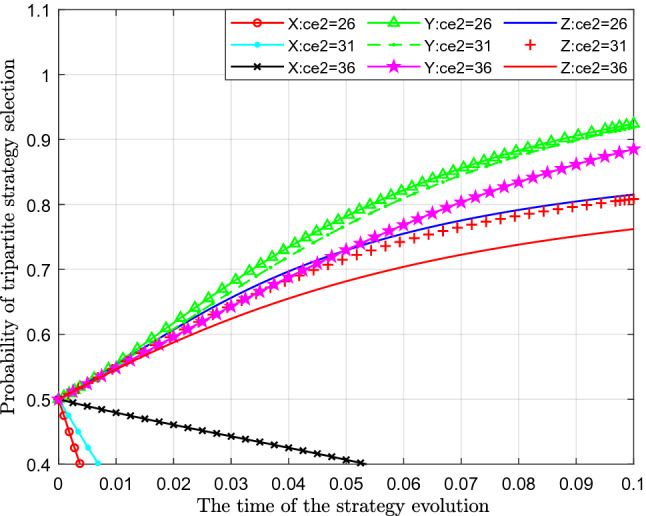
Effect of changes in excessive emission costs of sewage enterprises.

Figure 5 analyses the impact of the change in excessive emission cost of sewage enterprises on the evolutionary strategy. This paper assigns values of 26, 31, and 36 to $C_{e2}$ for analysis.

Figure 5 shows the effect of the change in excessive emission cost of sewage enterprises. From the figure, we can see that with a continuous increase in excessive emission cost, the probability of standard emission $X$ represented by separate colour curves also increases, and its probability gradually approaches 1. At the same time, the time the probability reaches 1 becomes increasingly shorter. From the perspective of the probability of public participation in the supervision of sewage enterprise $Y$, with an increase in excessive emission costs, the probability of public participation in supervision decreased significantly, which is shown as the curve moving downwards in the figure. From the perspective of the impact on the probability of government strict supervision $Z$, with a continuous increase in the cost of excessive emissions, the probability of government strict supervision also gradually decreases, which shows that with the gradual increase in the cost of excessive emissions of sewage enterprises, the probability of standard emissions gradually increases, and the probability of government strict supervision gradually decreases.

### Effect of changes in economic compensation $$ S_{P}$$ obtained from sewage enterprises

**Figure 6:**
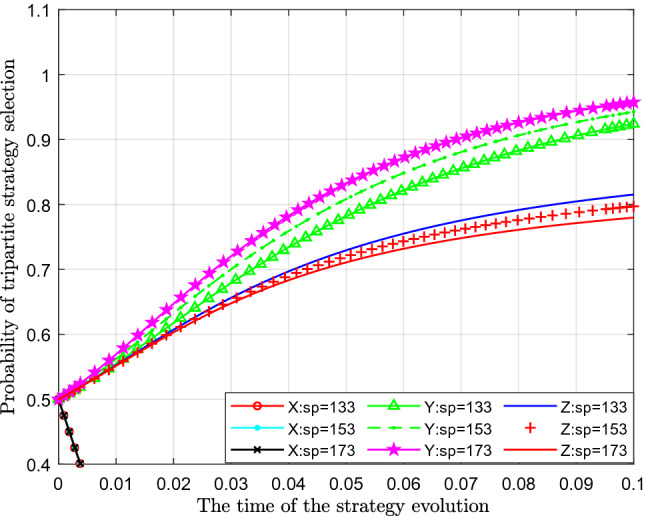
Effect of changes in economic compensation obtained from sewage enterprises.

Figure 6 analyses the impact of the change in economic compensation $$ S_{P}$$ obtained from sewage enterprises on the strategy. This paper assigns values of 133, 153, and 173 to $$ S_{P}$$ for analysis.

Figure 6 shows the effect of the change in economic compensation $$ S_{P} { }$$ obtained from sewage enterprises. From the figure, we can see that with a continuous increase in economic compensation $$ S_{P}$$ for the losses suffered by the public, the change in the probability of standard emissions $$X$$ represented by different colour curves has not changed significantly, which is almost negligible. From the perspective of the probability of public participation in supervision $$Y$$, with a continuous increase in economic compensation $$ S_{P}$$, the probability of public participation in supervision has increased slightly, which can be seen in the figure that the curves move upwards by a large margin. From the perspective of the impact on the probability of government strict supervision $$Z$$, with a continuous increase in economic compensation $$ S_{P}$$, the probability of government strict supervision also gradually decreases. With a continuous increase in economic compensation to the public, the probability of the public participating in supervision gradually increases, and then the probability and necessity of strict supervision by the government gradually decreases.

### Effect of the fine on excessive emission $F_{e}$

**Figure 7:**
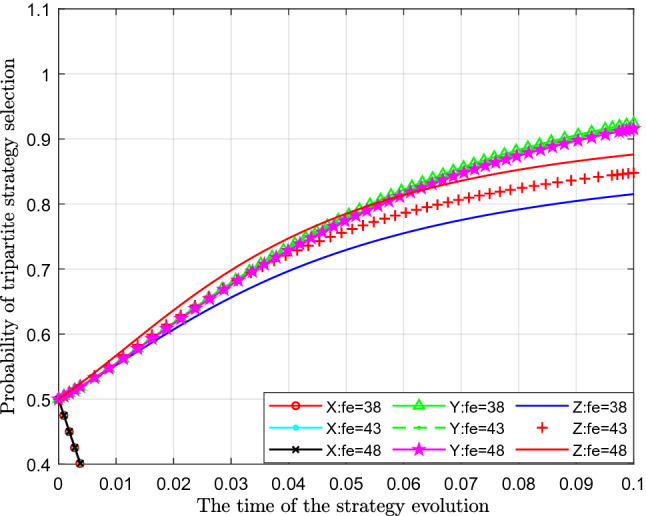
Effect of the change in penalty for excessive emissions.

Figure 7 analyses the impact of the change in excessive emission penalty on the evolutionary strategy. This article assigns values of 38, 43 and 48 to $$F_{e} $$ for analysis.

Figure 7 shows the effect of the change in penalty $$F_{e} $$ for excessive emissions. From the figure, we can see that with a continuous increase in the government's penalty for excessive emissions, the probability of standard emissions $$ X$$ represented by different colour curves does not change significantly, which can be almost ignored. From the perspective of the probability of public participation in supervision $$Y$$, with an increase in government fines for excessive emissions, the probability of public participation in supervision decreases, which is shown as the curve in the figure. From the perspective of the impact on the probability of strict supervision $$Z$$, with continuous increase of the government's fines for excessive emission $$F_{e}$$, the probability of government's choosing of strict supervision has increased significantly, which shows that with gradual increase of fines for excessive emission, government's income has increased, and then the probability of government's choosing of strict supervision has gradually increased.

### Effect of the cost of public participation in supervision $C_{p}$

**Figure 8:**
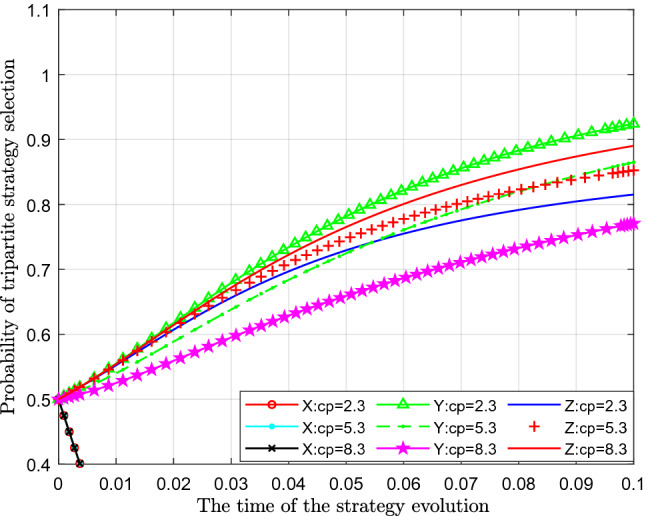
Effect of the cost of public participation in supervision.

Figure 8 analyses the impact of changes in the cost of public participation in supervision on the strategy. This paper assigns values of 2.3, 5.3 and 8.3 to $C_{p}$ for analysis.

Figure 8 shows the effect of the cost of public participation in supervision $C_{p}$. From the figure, we can see that with a continuous increase in the cost of public participation in the supervision of sewage enterprise $C_{p}$, the probability of standard emissions $X$ represented by separate colour curves hardly changes. From the perspective of the probability of public participation in supervision $Y$, with an increase in the cost of public participation in supervision $C_{p}$, the probability of public participation in supervision has decreased significantly, which can be seen in the figure that the curve moves downwards very clear. In terms of the impact on the probability of strict supervision $Z$, with a continuous increase in the cost of public participation in the supervision, the probability of strict supervision also gradually increases, which shows that with gradual increases in the cost of public participation in the supervision of sewage enterprises, the public is increasingly reluctant to participate in supervision. At this time, the necessity of strict supervision by the government was revealed, and then the probability of strict supervision by the government gradually increased.

### Effect of the change in reward for the public's participation in supervision $R_{P}$

**Figure 9:**
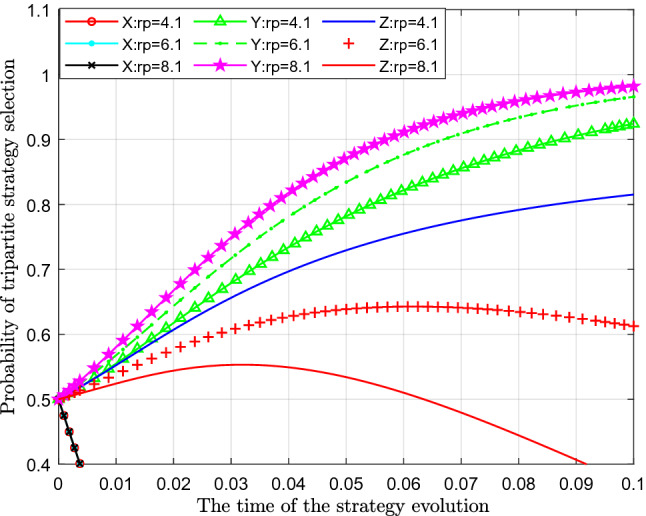
Effect of the change in reward on the public's participation in supervision.

Figure 9 analyses the impact of the change in reward for the public's participation in supervision $R_{P} { }$ on the evolutionary strategy. This paper assigns values of 4.1, 6.1 and 8.1 to $R_{P}$ for analysis.

Figure 9 shows the effect of the change in reward for the public's participation in supervision $$R_{P}$$. From the figure, we can see that with a continuous increase in the government's reward for the public's participation in supervision $$ R_{P}$$, the probability of standard emissions $$X$$ represented by different colour curves hardly changes. From the perspective of the probability of public participation in supervision $$Y$$, with an increase in the reward for the public participating in supervision $$R_{P}$$, the probability of public participation in the supervision of sewage enterprises also increases significantly, which can be seen in the figure that the curves increase significantly. In terms of the impact on the probability of strict supervision $$Z$$, with continuous increase of reward for the public's participating in supervision $$R_{P }$$, the probability of government's choosing of strict supervision has decreased significantly, which shows that with gradual increase of reward for the public's participating in supervision, government's cost has gradually increased, so government's willingness to reward public's participation in supervision has gradually declined.

### Effect of the change of strict supervision cost $C_{g1}$

**Figure 10:**
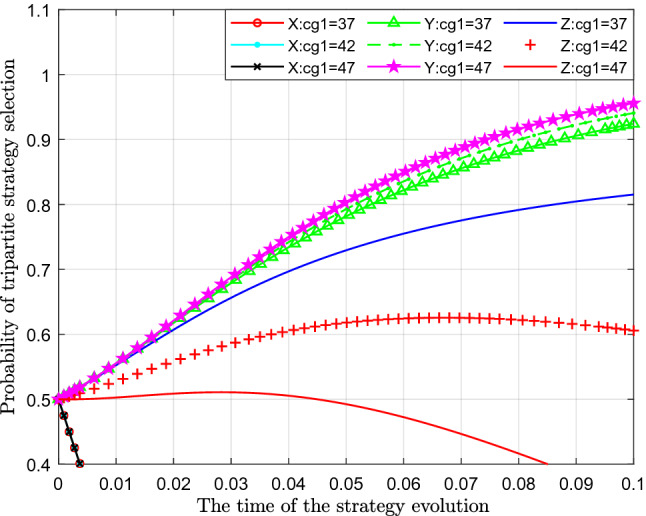
Effect of cost change of strict governmental regulation.

Figure 10 analyses the impact of changes in the cost of strict supervision $C_{g1}$ on the evolutionary strategy. This paper assigns values of 37, 42 and 47 to $C_{g1} { }$ for analysis.

Figure 10 shows the effect of the cost change of strict governmental regulation $C_{g1}$. From the figure, we can see that with a continuous increase in the cost of the government's strict supervision $C_{g1}$, the probability of standard emissions $X$ represented by different colour curves hardly changes. From the perspective of the probability of public participation in supervision, with the increase in the cost of strict supervision by the government, the probability of public participation in supervision $Y$ also increased slightly, which can be seen in the figure that the curves moved slightly upwards. From the impact on the probability of strict supervision $Z$, with continuous increase of the cost of strict supervision $C_{g1}$, the probability of government's choosing of strict supervision has decreased significantly, which shows that with gradual increase of the cost of strict supervision $C_{g1}$, the cost of government has gradually increased, and then the willingness and probability of government to implement strict supervision has gradually decreased.

### The effect of changes in the cost of loose supervision

**Figure 11:**
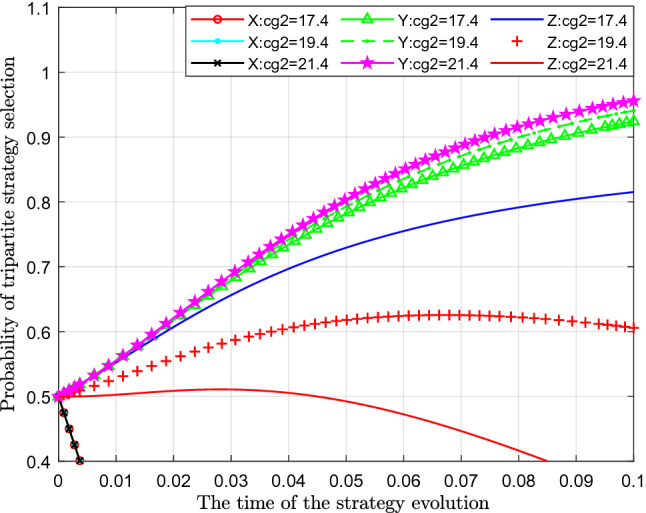
Effect of cost change of loose regulation by the government.

Figure 11 analyses the impact of changes in the cost of loose regulation by the government on the tripartite evolutionary game strategy. This paper assigns values of 17.4, 19.4 and 21.4 to $C_{g2} { }$ for analysis.

Figure 11 shows the effect of the cost change of the government's loose regulation. From the figure, we can see that with a continuous increase in the cost of government loose regulation $C_{g2}$, the probability of standard emissions $X$ represented by different colour curves hardly changes. From the perspective of the probability of public participation in supervision $Y$, with the increase in the cost of loose supervision by the government $C_{g2} { }$, the probability of public participation in supervision also increased slightly, which can be seen in the figure as the curves moved slightly upwards. From the perspective of the impact on the probability of strict supervision $Z$, with continuous increase of the cost of loose supervision $C_{g2} { }$, the probability of government's choosing of strict supervision has decreased significantly, which shows that with gradual increase of the cost of loose supervision $C_{g2} { }$, the cost of government has gradually increased, and then the willingness and probability of the government to implement strict supervision has gradually decreased.

### Effect of changes in potential economic losses caused by strict government supervision $L$

**Figure 12:**
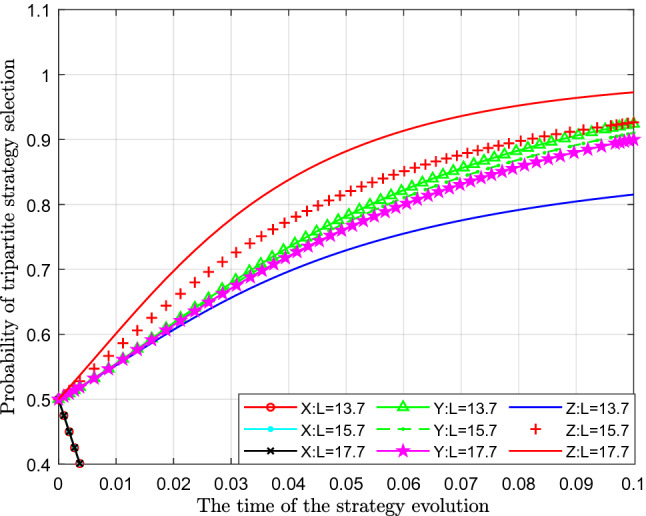
Effects of changes in potential economic losses caused by strict government regulation.

Figure 12 analyses the impact of changes in potential economic losses caused by strict government supervision on the evolutionary strategy. This paper assigns values of 13.7, 15.7 and 17.7 to $L$ for analysis.

Figure 12 shows the effect of the change in potential economic loss caused by strict governmental supervision $$L$$. From the figure, we can see that with a continuous increase in potential economic loss caused by strict governmental supervision $$ L$$, the probability of standard emissions $$X$$ represented by separate colour curves hardly changes. From the perspective of the probability of public participation in supervision $$ Y$$, with an increase in potential economic losses caused by the strict supervision of the government, the probability of public participation in supervision also increased slightly, which shows that there is a slight upward movement of the curves in the figure. From the perspective of the impact on the probability of strict government supervision $$Z$$, with continuous increase of potential economic losses caused by strict supervision $$L$$, the probability of government's choosing of strict supervision has decreased significantly, which shows that with gradual increase of potential economic losses caused by strict supervision, government's willingness and probability to implement strict supervision has gradually decreased.

### Effect of punishment change of superior government given to subordinate government for the absence of supervision $F_{g}$.

**Figure 13:**
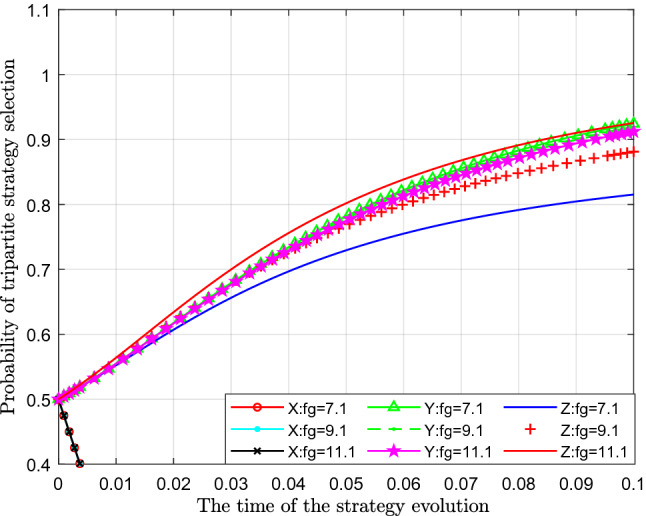
Effect of punishment change for absence of supervision.

Figure 13 analyses the impact of the change in the punishment of the superior government to the subordinate government with an absence of supervision on the evolutionary strategy. This paper assigns values of 7.1, 9.1 and 11.1 to $F_{g}$ for analysis.

Figure 13 shows the effect of the change in punishment for the absence of supervision $$F_{g}$$. From the figure, we can see that with a continuous increase in punishment for the absence of supervision $$ F_{g}$$, the probability of standard emissions $$ X$$, represented by separate colour curves, almost does not change. From the perspective of the probability of public participation in supervision $$Y$$, with the increase in punishment for the absence of supervision $$ F_{g}$$, the probability of public participation in supervision has decreased slightly, which can be seen in the figure that there is a slight downwards movement of the curves, reflecting a supervision substitution effect. From the perspective of the impact on the probability of strict supervision by the government $$Z$$, with continuous increase of the punishment of for the absence of supervision $$ F_{g}$$, the probability of government's choosing of strict supervision has increased significantly, which shows that with gradual increase of the punishment for the absence of supervision $$ F_{g}$$, the probability of government's strict supervision has gradually increased.

## Conclusions and policy implications

This paper, through analysis of the tripartite evolutionary game of sewage enterprises, local governments and the public, established replicator dynamic equations of the three players and analysed the existence and stability of the stable state. On this basis, parameters of the replicator dynamic equation are assigned, and the influence of the change of parameters on the equilibrium strategies of all parties involved in the game is analysed. By means of a two-dimensional diagram, this paper analysed the impact of changes in factors such as government punishment for excessive emissions and rewards to the people participating in the supervision of sewage enterprises on the strategies of all parties involved in the game.

The government's increase in the punishment of excessive emissions will help to improve the enthusiasm of sewage enterprises to meet the standard. With an increase in government punishment, the government's willingness to choose a strict supervision strategy will increase. The government's reward for public participation in the supervision of sewage enterprises must be greater than the increased cost of public participation in supervision so that the public will choose to supervise sewage enterprises. Reducing the cost of standard emissions, increasing the reward for public participation in the supervision of sewage enterprises, reducing the cost of strict supervision by the government, increasing the punishment for local government inactions, and reducing the economic loss of strict supervision are all measures to improve the probability of standard emissions.

This paper considers the game only among sewage enterprises, local governments and the public; it does not consider other possible stakeholders and does not consider the game order or the impact of initial value of parameters on the game results. Therefore, our future research direction is to introduce more stakeholders, build more game models, conduct dynamic and repeated games, study the influence mechanism of various factors on the game, and obtain more innovative results.

## Data Availability

The datasets used or analyzed during the current study are available from the corresponding author on reasonable request.
